# Bony Apprehension Test for Identifying Bone Loss in Patients With Traumatic Anterior Shoulder Instability: A Validation Study

**DOI:** 10.1177/03635465221085673

**Published:** 2022-03-31

**Authors:** Michael James, Cory A. Kwong, Kristie D. More, Justin LeBlanc, Ian K.Y. Lo, Aaron J. Bois

**Affiliations:** * Section of Orthopaedic Surgery, Department of Surgery, Cumming School of Medicine, University of Calgary, Calgary, Canada; † Sport Medicine Centre, University of Calgary, Calgary, Canada; ‡ McCaig Institute for Bone and Joint Health, University of Calgary, Calgary, Canada; Investigation performed at the McCaig Institute for Bone and Joint Health, University of Calgary, Calgary, Canada

**Keywords:** shoulder instability, apprehension test, bipolar bone loss, physical examination

## Abstract

**Background:**

The presence of bone loss has important implications for the surgical treatment of patients with recurrent shoulder instability. The bony apprehension test (BAT) is a physical examination maneuver that was designed to improve specificity from the anterior apprehension test (AAT) in detecting critical bone loss.

**Purpose:**

The purpose of this study was to compare the BAT with the AAT and relocation test based on their abilities to predict critical bone loss. Several well–described criteria were utilized to capture critical (≥25%) and subcritical (≥13.5%) glenoid defects, as well as Hill-Sachs defects (≥19%). The ability of the BAT to predict bipolar bone loss was also assessed, as indicated by engaging Hill-Sachs defects and off–track lesions.

**Study Design:**

Cohort study (diagnosis); Level of evidence, 1.

**Methods:**

The study cohort included patients ≥18 years of age who were scheduled to undergo arthroscopic stabilization for traumatic anterior shoulder instability. Notable exclusion criteria included multidirectional shoulder instability, connective tissue disorders, and workers’ compensation or litigation cases. Patients underwent physical examination immediately before surgery by the treating surgeon (ie, before the induction of anesthesia). Critical glenoid and humeral bone defects were measured on preoperative computed tomography scans. Hill-Sachs engagement and on- or off–track determination of bone loss were assessed arthroscopically and via computed tomography, respectively.

**Results:**

A total of 52 patients were included in the study. In cases of subcritical glenoid bone loss (≥13.5%) and critical Hill-Sachs defects (≥19%), the BAT had good and fair specificity (82% and 72%, respectively) but poor sensitivity (40% and 39%). The BAT also had poor sensitivity (0%), specificity (67%), and positive predictive value (0%) for higher percentages of glenoid bone loss (≥25%). When engaging Hill-Sachs lesions were assessed, the BAT had excellent specificity (94%) and positive predictive value (94%) but poor sensitivity (43%) and negative predictive value (44%). Furthermore, the BAT performed poorly at predicting off–track humeral lesions. The AAT demonstrated 100% sensitivity and 0% specificity in detecting all measures of bone loss.

**Conclusion:**

The BAT performed poorly at identifying subcritical and critical bone loss and was not found to have any clinical value. Future work is needed to identify a physical examination test that could complement advanced imaging for preoperative assessment of critical bone loss.

The shoulder remains the most commonly dislocated major joint, with recurrence rates decreasing with age.^9,14^ Recurrent anterior shoulder instability is known to result in bony lesions of the anteroinferior glenoid (ie, erosive glenoid bone loss and/or a bony Bankart lesion) and posterolateral humeral head (ie, Hill-Sachs defect), which dramatically affect the prognosis and success of isolated soft tissue repairs.^3,18^ In patients with glenoid lesions comprising ≥25% of the glenoid surface or with Hill-Sachs lesions that engage the glenoid rim, recurrence rates as high as 67% have been reported after soft tissue repairs alone and support the indication for bony augmentation procedures such as the Latarjet.^1,3,10^

An osseous lesion involving ≥25% of the glenoid surface has been traditionally described as “critical” glenoid bone loss,^20^ although concepts such as “borderline” (20%)^15^ and “subcritical” (13.5%)^19^ have more recently been proposed. However, the interaction of glenoid and concomitant humeral defects may be the most important factor to consider when managing patients with bipolar defects.^13^ Thus, the concepts of engaging^3^ and on- and off–track lesions^8^ have gained substantial attention in recent years to better understand the interaction of bony lesions on either side of the joint.

Preoperative evaluation of patients with anterior shoulder instability consists of clinical and radiographic examinations. The bony apprehension test (BAT) is one described method for detecting critical bone loss preoperatively.^5,17^ In 2004, Miniaci and Gish^17^ reported physical examination findings of apprehension with the arm in a position of ≤45° of abduction and ≤45° of external rotation in patients with large engaging Hill-Sachs lesions undergoing revision surgery. In 2008, Bushnell et al^5^ termed this lower abduction–external rotation position of apprehension the BAT, an adaptation of the traditional anterior apprehension test (AAT). Bushnell et al evaluated the BAT by its ability to predict the presence of a critical bony lesion, defined as glenoid deficiency ≥25% and/or an engaging Hill-Sachs lesion ≥2 cm in length at the time of diagnostic arthroscopy. Of the 29 patients in the original study, 8 had substantially large bony lesions (ie, requiring reconstruction), and the BAT revealed a sensitivity of 100% and specificity of 86%,^5^ which is far more accurate than most shoulder physical examination tests.^11^ To our knowledge, there have not been any prospective studies validating the BAT since the pilot study performed by Bushnell et al.

This study aimed to analyze the ability of the BAT to reliably predict clinically relevant bony lesions in patients with recurrent traumatic anterior instability. The BAT was also compared with the standard AAT and relocation test in their ability to predict glenoid and humeral bone loss, engaging Hill-Sachs lesions, and on- and off–track lesions using more precise measurement techniques and definitions of critical bone loss. We hypothesized that the BAT would have comparable sensitivity and better specificity than the AAT and relocation test in predicting clinically important bony defects.

## Methods

### Study Design

The study was a prospective, single–blinded cohort design that was approved by the University of Calgary Research Ethics Board (REB17-0575). Eligible patients presenting to 1 of the 3 surgeon investigators (J.L., I.K.Y.L., A.J.B.) were offered the opportunity to participate. Eligible patients underwent initial preoperative physical examination at the time of study enrollment by the consenting surgeon and before anesthetic induction on the day of surgery by the same surgeon. Included were patients aged ≥18 years who were scheduled to undergo arthroscopic stabilization for recurrent traumatic anterior shoulder instability (defined as ≥2 dislocations). Patients then underwent preoperative 3–dimensional computed tomography (3D-CT) imaging. Exclusion criteria were as follows: multidirectional or posterior shoulder instability, patients underging open stabilization procedures or revision surgery, connective tissue disorders, workers’ compensation or litigation cases, and non–English speaking.

### Primary Outcomes

The primary outcomes were the sensitivity, specificity, positive predictive value, and negative predictive value of the BAT, AAT, and relocation test in predicting multiple variables of glenohumeral bone loss.

### Sample Size

The sample size was based on the study by Bushnell et al,^5^ with an estimated 27.6% prevalence of critical bone loss among patients with recurrent anterior shoulder instability. Assuming an actual sensitivity of 90% (vs 100% in the Bushnell et al pilot study), we had 85.8% power to detect a clinically useful test via a 1–sided exact binomial test at an alpha of .05. Assuming an actual specificity of 80% (vs 86% in the Bushnell et al pilot study), we had 84.3% power to detect a clinically useful test. The resulting sample size was 50.

### Physical Examination Maneuvers

#### Bony Apprehension Test

**Figure 1.**
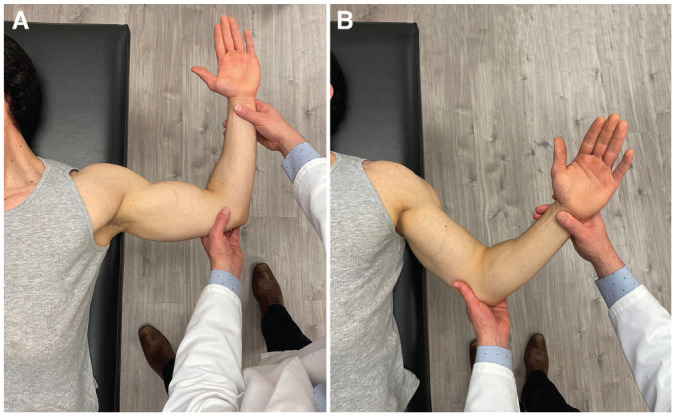
(A) Anterior apprehension test. (B) Bony apprehension test. For each test, the patient is positioned supine with the scapula stabilized on the examining table.

Each patient was positioned in the supine position with the scapula stabilized. For the standard BAT, the affected shoulder was placed in 45° of abduction and 45° of external rotation. A positive test result required reproduction of the patient's apprehension symptoms (Figure 1). Pain alone was considered a negative test result.

#### Anterior Apprehension Test

With the patient positioned in the supine position, the affected shoulder was placed in 90° of abduction and maximum external rotation by the examiner. A positive test result required reproduction of the patient's apprehension symptoms. Pain alone was considered a negative test result.

#### Relocation Test

If the AAT result was positive, a posteriorly directed force was placed on the midupper humerus. A positive test result required resolution of the apprehension symptoms elicited from the AAT.

#### Identification of the Position of Apprehension

**Figure 2.**
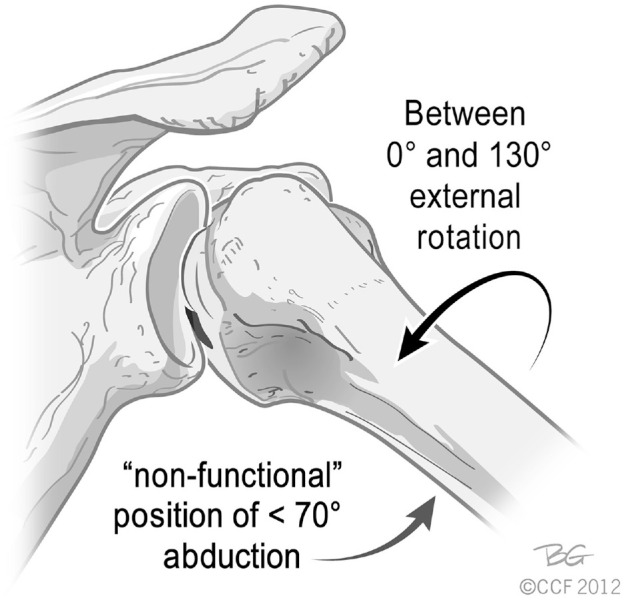
Nonfunctional position of engaging Hill-Sachs lesion. Adapted with permission from Burkhart and De Beer.^3^ Also adapted from Bois and Miniaci.^2^ Reprinted with permission from the Cleveland Clinic Center for Medical Art and Photography 2012-2022. All rights reserved.

After the BAT, AAT, and relocation maneuvers, each examiner returned the patient's shoulder to 0° of abduction and 0° of external rotation. The examiner then slowly increased the abduction angle, after the shoulder was initially placed in external rotation, until a sensation of apprehension was elicited. The degrees of abduction and external rotation at the onset of apprehension were documented in an attempt to assess the functional and nonfunctional (≤70°) positions of the shoulder during apprehension (Figure 2).^3^

### Data Collection

#### Clinical Parameters

Before the start of the study, the research team reviewed the technique required to perform the BAT, AAT, and relocation test to ensure accuracy and consistency. On the day that patients consented for surgery (ie, initial visit), the surgeon performed and recorded the results of the 3 tests—the BAT, AAT, and relocation test—as well as the patient's precise position of apprehension. On the day of surgery, the surgeon performed the same 3 tests with the patient awake, and the results were used for our primary analysis. CT scan images and/or measurements were not reviewed before clinical examination on the day of surgery.

Intraoperatively, the surgeon proceeded with a diagnostic arthroscopy, systematically examining all shoulder joint structures. Of note, 2 surgeons utilized beach–chair positioning (J.L. and A.J.B.), and 1 surgeon used the lateral decubitus position (I.K.Y.L.) for all procedures. After completion of the diagnostic arthroscopy, an anterior reconstruction was performed addressing all necessary pathology to ensure shoulder stability. All surgical findings were recorded on a surgical data collection form.

#### Radiographic Parameters

##### Computed Tomography

All CT data were collected using unique patient identifiers to ensure surgeon blinding. All CT measurements were completed postoperatively, and such data were therefore not available to the treating surgeon on the day of surgery.

##### Glenoid Bone Loss Measurements

**Figure 3.**
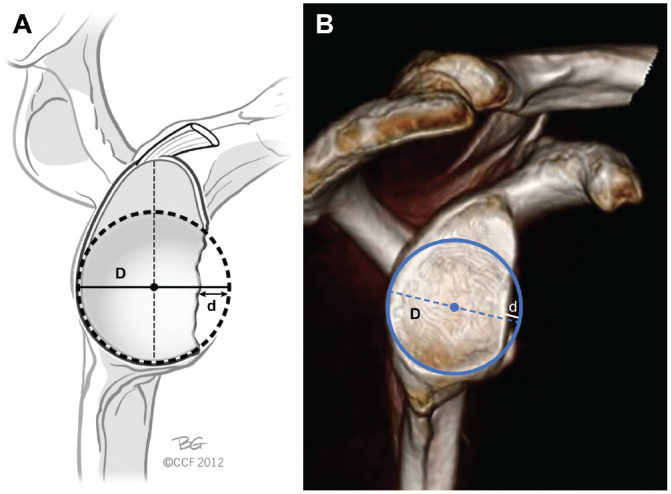
(A) Illustration and (B) enface view of the glenoid fossa on 3–dimensional computed tomography reconstruction demonstrating the technique used for measuring glenoid bone loss utilizing the width of glenoid defect (*d*) and the diameter of the circle of best fit (*D*). Adapted from Bois and Miniaci.^2^ Reprinted with permission from Cleveland Clinic Center for Medical Art and Photography 2012-2022. All rights reserved.

A 3D-CT reconstruction of the isolated scapula was oriented to give an en face view of the glenoid, and a circle of best fit was determined. The width of the glenoid defect (*d*) was divided by the diameter of the circle of best fit (*D*) to yield the percentage of glenoid bone loss (Figure 3).^4^ The 3 investigating surgeons performed measurements on 2 occasions, 6 weeks apart, after completion of the physical examination and arthroscopic data collection. An average of the resultant 6 measurements was used for our analysis.

##### Humeral Bone Loss Measurements

**Figure 4.**
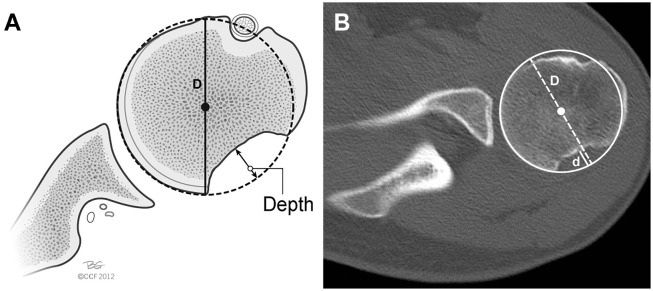
(A) Illustration and (B) axial computed tomography image demonstrating the technique used for measuring Hill-Sachs lesions utilizing the depth of the Hill-Sachs lesion (*d*) and humeral head diameter (*D*). Adapted from Bois and Miniaci.^2^ Reprinted with permission from Cleveland Clinic Center for Medical Art and Photography 2012-2022. All rights reserved.

The axial CT slice that demonstrated the maximum Hill-Sachs depth was chosen for measurement, and a circle of best fit encompassing the humeral head was identified. Axial slice selection was determined via consensus of the primary author (M.J.) and principal investigator (A.J.B.). The width of the Hill-Sachs defect (*d*) was divided by the diameter of the humeral head (*D*) to yield percentage bone loss (Figure 4).^7^ The same 2 investigators measured each scan twice to ensure agreement on maximum depth and appropriate circle of best fit.

##### On- vs Off-track Determination

**Figure 5.**
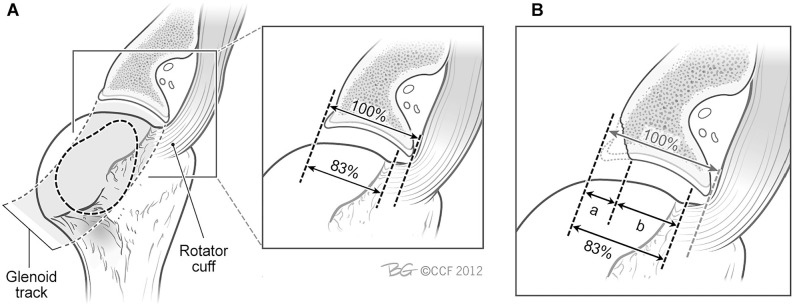
Glenoid track concept. (A) In extremes of external rotation and abduction, the glenoid displaces the cuff tendon close to its footprint, creating a glenoid track that is close to 83% of the intact glenoid width. (B) When a glenoid defect exists, the defect width is subtracted from the 83% width obtained from the normal glenoid to calculate the true glenoid track width. Adapted with permission from Yamamoto N, Itoi E, Abe H, et al.^24^ Also adapted from Bois and Miniaci.^2^ Reprinted with permission from Cleveland Clinic Center for Medical Art and Photography 2012-2022. All rights reserved.

Preoperative 3D-CT scans were assessed using the method described by Di Giacomo et al.^8^ The 3 investigating surgeons performed measurements on 2 occasions, 6 weeks apart. This consisted of estimating the native glenoid width (*D*) to determine the amount of bone loss (*d*); the glenoid track was calculated as *D* (0.83) –*d* (Figure 5). The Hill-Sachs interval was determined by measuring the width of both the Hill-Sachs lesion and bone bridge on a 3D-CT reconstruction of the isolated humerus. The bone bridge is the distance between the medial rotator cuff footprint and the lateral margin of the Hill-Sachs lesion. The Hill-Sachs interval is equal to the Hill-Sachs lesion + the bone bridge (ie, the distance from the medial border of the Hill-Sachs lesion to the medial border of the rotator cuff footprint). If the glenoid track width was greater than the Hill-Sachs interval, then the lesion was defined as on–track, whereas a glenoid track less than the Hill-Sachs interval was defined as off–track.^8^

#### Intraoperative Parameters

##### Dynamic Arthroscopic Assessment of Engaging vs Nonengaging Hill-Sachs

Before the soft tissue repair, a dynamic assessment of engagement was performed. For standardization purposes and to ensure that the point of engagement was most easily observed arthroscopically, we first placed the shoulder in maximal external rotation and then slowly abducted the shoulder to assess if the Hill-Sachs lesion engaged the anterior glenoid rim. This was similar to the clinical assessment of the position of apprehension. The determination of engagement was made via visualization alone (ie, confirmation that the Hill-Sachs defect engaged the anterior glenoid rim). Visualization of engagement was confirmed while viewing from a standard posterior portal and/or anterosuperolateral portal. In cases of engagement, the position of abduction and external rotation was recorded. All surgeons were blinded from the CT parameter results at the time of the arthroscopic evaluation.

### Data Analysis

The predictive utility of the BAT, AAT, and relocation test was computed for assessing various measures of bone loss: subcritical glenoid bone loss (≥13.5%), critical Hill-Sachs defects (≥19%), critical glenoid bone loss (≥25%), and engaging Hill-Sachs and off–track lesions. Analysis was also performed to assess whether abduction ≤70° was predictive of bone loss and whether any combination of abduction and external rotation was reliably predictive of bone loss. Predictive utility measures included sensitivity, specificity, positive predictive value, and negative predictive value. Logistic regression models were fit to predict each bone loss measure from the position of apprehension on abduction and external rotation, as well as their interaction term. Model fit was assessed using the Hosmer and Lemeshow goodness–of–fit test.^6^

### Reliability Analysis

Interrater reliability was determined by comparing the surgeon's physical examination with the surgical assistant's clinical examination (or equivalent) on the day of surgery. Intrarater reliability was determined by comparing the surgeon's physical examination during the initial clinical assessment with that performed on the day of surgery. All study investigators were trained in performing the pertinent physical examination tests. The clinical examinations were performed independently, and each examiner had no knowledge of the other's results. The intra- and interrater reliabilities were measured using the kappa statistic and percentage agreement. The kappa statistic,^16^ which was developed by Jacob Cohen, was interpreted as follows: values ≤0 indicate no agreement; 0.01 to 0.20, none to slight; 0.21 to 0.40, fair; 0.41 to 0.60, moderate; 0.61 to 0.80, substantial; and 0.81 to 1.00, almost perfect. Analyses were performed using SAS version 9.4 (SAS Institute Inc., Cary, NC, USA).

## Results

**Figure 6.**
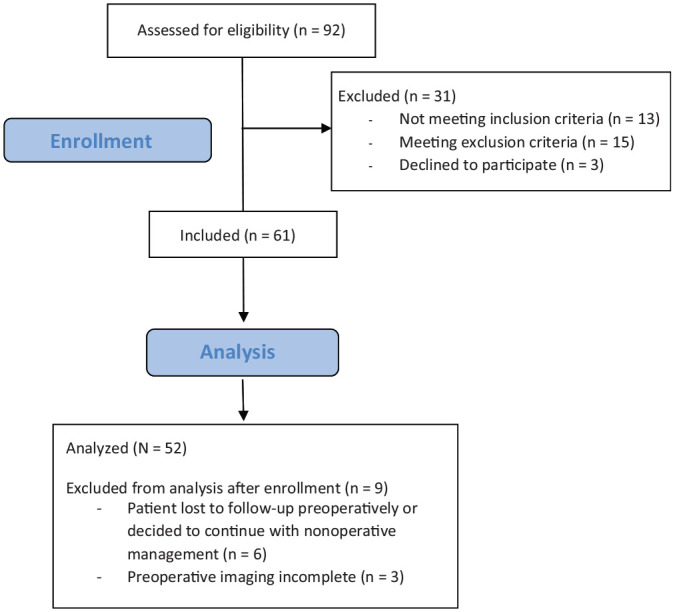
Flowchart demonstrating patient eligibility, recruitment, and analysis.

A total of 92 patients were assessed for study eligibility. Nine were excluded after initial study enrollment, and 52 were included in the final analysis: 12 women (23%) and 40 men (77%) (Figure 6). The average age at the time of surgery was 31 years (range, 18-49 years) (Table 1). The mean number of dislocations was 12 (SD, 10; range, 2-45). The prevalence of bone loss parameters is listed in Table 2.

**Table 1:** Patient Characteristics, Number of Dislocations and Bone Loss Parameters (52 Patients)^*a*^

Age, y	30.6 (18-49)
Sex, No. (%)	
Male	40 (77)
Female	12 (23)
No. of dislocations	12 ± 10 (2-45)
Defect size, %	
Hill-Sachs	16 ± 6
Glenoid	14 ± 6

a Values are presented as mean ± SD (range) unless noted otherwise.

**Table 2:** Prevalence of Bone Loss (52 Patients)

Clinical Parameter	No. (%)
Glenoid bone loss	
≥25%	3 (5.8)
≥13.5%	30 (57.7)
Hill-Sachs ≥19%	13 (25.0)
Combined bone loss	
≥30%	25 (48.1)
≥35%	11 (21.2)
Engaging Hill-Sachs	35 (67.3)
Off-track lesion	33 (73.3)^*a*^

a Out of 45 patients.

In cases of subcritical glenoid bone loss (≥13.5%) and critical Hill-Sachs defects (≥19%), the BAT had good and fair specificity (82% and 72%, respectively) but poor sensitivity (40% and 39%, respectively). The BAT had poor sensitivity (0%) and specificity (67%) for predicting glenoid bone loss ≥25%. However, there were only 3 (5.8%) patients in our cohort with this magnitude of bone loss. When engaging Hill-Sachs lesions were assessed, the BAT had excellent specificity (94%) and positive predictive value (94%) but poor sensitivity (43%) and negative predictive value (44%). Furthermore, the BAT performed poorly at predicting off–track humeral lesions, with a sensitivity and specificity of 50% and 76%, respectively. Analysis of abduction angle ≤70° in any position of external rotation had similarly low accuracy regardless of bone loss measure (Table 3). The AAT and relocation test results were positive in all 52 patients, giving a sensitivity of 100% and a specificity of 0% in predicting any degree of bone loss, engaging Hill-Sachs lesions, and off–track lesions (Table 4).

**Table 3:** Predictive Ability: BAT and Abduction ≤70°^*a*^

	BAT				Abduction ≤70°			
Clinical Parameter	SN	SP	PPV	NPV	SN	SP	PPV	NPV
Glenoid bone loss								
≥25%	0	67.3	0	91.7	50.0	20.0	4.0	85.7
≥13.5%	40.0	81.8	75.0	50.0	73.9	11.1	68.0	14.3
Hill-Sachs ≥19%	38.5	71.8	31.3	77.8	100	29.2	32.0	100
Combined bone loss ≥30%	40.0	77.8	62.5	58.3	72.2	14.3	52.0	28.6
Engaging Hill-Sachs	42.9	94.1	93.8	44.4	80.0	50.0	87.5	4.2
Off-track lesion	50.0	75.8	42.9	80.6	70.0	25.0	36.8	57.1

a Values are presented as percentages. BAT, bony apprehension test; NPV, negative predictive value; PPV, positive predictive value; SN, sensitivity; SP, specificity.

**Table 4:** Predictive Ability: AAT and Relocation Maneuver^*a*^

	AAT			Relocation Test		
Clinical Parameter	SN	SP	PPV	SN	SP	PPV
Glenoid bone loss						
≥25%	100	0	5.8	100	0	5.8
≥13.5%	100	0	57.7	100	0	57.7
Hill-Sachs ≥19%	100	0	25.0	100	0	25.0
Combined bone loss ≥30%	100	0	48.1	100	0	48.1
Engaging Hill-Sachs	100	0	67.3	100	0	67.3
Off-track lesion	100	0	26.7	100	0	26.7

a Values are presented as percentages. Negative predictive value was not applicable. AAT, anterior apprehension test; PPV, positive predictive value; SN, sensitivity; SP, specificity.

A regression analysis utilizing binary logistic models of the exact position of apprehension failed to identify any combination of abduction and external rotation that reliably predicted any measure of bone loss, engaging Hill-Sachs lesions, or off–track lesions. Of note, the binary logistic models had adequate fit according to the Hosmer and Lemeshow goodness–of–fit test.^6^

Reliability analysis showed fair intra- and interrater reliabilities of the BAT based on the kappa statistic (Table 5). For the AAT and relocation intra- and interrater reliabilities, kappa was less useful given a larger amount of missing data, but percentage agreement was high (>80%) for both maneuvers.

**Table 5:** Reliability Statistics: Physical Examination Maneuvers

	Intrarater		Interrater	
Test	Kappa	Agreement, %	Kappa	Agreement, %
Bony apprehension	0.35	68.2	0.25	66.7
Anterior apprehension	0	98.0	0	91.2
Relocation	0	86.7	−0.05	81.3

## Discussion

Our study demonstrated that the BAT had unacceptably poor sensitivity and specificity to be considered a useful physical examination maneuver in the setting of recurrent traumatic anterior instability. It also failed to reproduce the results of the Bushnell et al^5^ pilot study in which the BAT showed 100% sensitivity and 86% specificity in detecting significant bony lesions.^5^ This disparity may be due to a variety of factors, many of which were directly referenced in their study.

First, Bushnell et al^5^ included patients undergoing revision, who represented 38% of their “significant bone loss” cohort (n = 3/8). We opted to exclude patients undergoing revisions since their inclusion would confound our analysis of the BAT. We also excluded patients with multidirectional instability, connective tissue disorders, and workers’ compensation cases for similar reasons. Bushnell et al did not explicitly address the inclusion or exclusion of patients with multidirectional instability, connective tissue disorders, and workers’ compensation cases or the number of dislocations before surgical treatment. It is possible that Bushnell et al included patients with first–time dislocations whereas our study comprised only those patients with recurrent instability (≥2 dislocations), which may have affected the results.

Second, our cohorts differed considerably in size (52 vs 29), as well as prevalence of critical bone loss. Bushnell et al^5^ included 6 patients (21%) with glenoid bone loss ≥21% (average, 36%; range, 25%-50%), while our cohort had just 3 such patients (5.8%). This may be due to the different patient populations studied but may also be related to the different techniques used to calculate bone loss. While Bushnell et al used arthroscopic measurements rounded to the nearest 5% to calculate glenoid bone loss, our study used CT measurements rounded to the nearest percentage. Furthermore, our glenoid measurements were averaged across 3 examiners and conducted on 2 occasions. The latter likely contributed to more precise measurements, but the accuracy of arthroscopic versus CT measurement of glenoid bone loss is less clear.^23^ Regardless, the sensitivity of the BAT in predicting glenoid bone loss ≥25% differed greatly between our study and that of Bushnell et al (0% vs 100%).

The second criterion of significant bone loss in the Bushnell et al^5^ study was a Hill-Sachs defect with “at least 2 cm of engagement length” measured arthroscopically. This represented 7% (2/29) of their cohort, wherein both patients required reconstructive procedures to address the large humeral head lesion. The rationale for using an engaging Hill-Sachs defect of at least 2 cm remains unclear, but it may have been chosen arbitrarily since measures of “critical” humeral bone loss were not well established. Our study included a Hill-Sachs defect ≥19% (relative to the humeral head diameter), as our measure of critical humeral–sided bone loss is in keeping with more recent biomechanical studies of bipolar bone loss.^22^ On the basis of this definition, 13 patients from our study cohort (25%) had critical humeral bone loss; however, sensitivity and specificity remained poor at 39% and 72%, respectively. In the original study by Miniaci and Gish,^17^ all 18 patients had substantially large Hill-Sachs defects (≥25% of articular arc circumference), and all had previous soft tissue repairs that failed (ie, revision cases). Yet, only 3 (17%) patients had glenoid bone loss (<20%; subcritical) and represented the only 3 patients with bipolar bone loss in their cohort. As our study did not utilize the articular arc length to determine the size of humeral head defects, it is possible that the size of humeral defects within our study cohort was not large enough to elicit apprehension at lower abduction angles. Our study also excluded revision cases in our patient cohort, and this variable alone may in part explain our study results.

One area in which our study concurred with that of Bushnell et al^5^ was in demonstrating poor specificity of the traditional AAT. As in the Bushnell et al study, 100% of our cohort had a positive AAT result, giving a sensitivity of 100% and specificity of 0% for all outcomes of interest. The addition of the relocation maneuver in our study added no benefit, as it was uniformly positive. This ceiling effect limited the utility of these tests for surgical management of instability with concomitant bone loss, a void that the BAT was designed to fill.

The rationale for implementing a physical examination maneuver with a lower abduction and external rotation angle (≤45°) was based on a study by Walia et al.^22^ Their study demonstrated that apprehension can occur below 90° of abduction and 90° of external rotation and was more typical in patients with critical bone loss. Our study attempted to improve upon these arbitrarily chosen angles by recording the exact combination of abduction and external rotation angles at which each patient first experienced apprehension. Our study also assessed a value of abduction between 45° and 90° (≤70° abduction; ie, “nonfunctional” position)^3^ to determine if this improved sensitivity as compared with the BAT while improving specificity as compared with the AAT and relocation maneuver. However, our binary logistic regression analyses could not identify any trends with regard to defining a more accurate combination of abduction and external rotation angles. Conversely, Godinho et al^12^ retrospectively analyzed correlations of the apprehension test at lower abduction angles (0°, 45°, and 90°) and found that they were positively correlated with off–track lesions and glenoid bone loss >13.5%. Yet, the authors noted several limitations within their study, specifically the retrospective nature and the lack of reliability analysis. Additionally, they did not directly evaluate the diagnostic accuracy of the apprehension test at 0°, 45°, and 90°, which makes direct comparison with our study findings problematic.

One of the limitations of our study is that a precise number of dislocations in between the initial clinic visit or CT scan and surgical date was not obtained. This was largely due to patient recall regarding when, if any, instability episodes had occurred. Thus, the bone loss measurements obtained from CT scans may not be representative of the bone loss assessed at the time of surgery. Furthermore, interval instability events may have influenced the clinical examination results on the day of surgery. This limitation is common among studies with dynamic pathology that compare preoperative imaging with intraoperative assessment.

Another limitation of our study and a possible explanation for our disparate results compared with those of the Bushnell et al study^5^ could extend from small inconsistencies in performing the BAT. This was demonstrated by the low kappa and percentage agreement upon analysis of intra- and interrater reliability. Despite standardized training and agreement among all study personnel in performing the BAT, a post hoc analysis revealed that subtle differences in technique may have existed between examiners that potentially influenced the precision of the BAT (eg, addition of shoulder extension as part of the maneuver). As is common in clinical practice, each examiner estimated the shoulder position of apprehension rather than using a measurement device such as a goniometer; therefore, the precise degree of shoulder position cannot be confirmed. Furthermore, rather than performing a continuous range of motion to identify the exact position at which patients experienced apprehension, a more precise analysis could have included discrete combinations of abduction and external rotation beyond 45°/45° and 90°/90°.

Subtle differences in uniformity in physical examination maneuvers are a common phenomenon across orthopaedic surgeons and allied professionals, who tend to make nuanced alterations to standard maneuvers based on clinical experience. While the physical examination often becomes subjugated owing to the evolution of advanced imaging modalities, the ubiquity of asymptomatic imaging findings and normal physiologic variants has placed the focus back on the need for physicians to obtain a thorough patient history and perform a proper disease–specific shoulder examination. Thus, standardizing physical examination techniques remains a vital part of clinical practice and research. Future research is required to assess the ability of clinical tests such as the anterior drawer and the load and shift in identifying critical bone loss given that both these tests demonstrate better specificity than the AAT in diagnosing traumatic shoulder instability.^21^ In addition, evaluating these maneuvers as predictors of critical bone loss in patients with first–time dislocation may have more clinical utility versus in those with recurrent instability, as the latter group will likely undergo advanced imaging regardless of physical examination findings.

The strengths of our study include our rigor in study methodology, such as sample size calculations and bone loss measurement, as well as the exclusion of patients undergoing revision surgery, those with multidirectional instability, and workers’ compensation cases, which led to a cleaner analysis of the BAT. The results of this study are also generalizable to all orthopaedic surgeons treating patients with anterior shoulder instability and are not limited to those practicing only in academic centers.

## Conclusion

Our study did not establish the BAT as a useful physical examination maneuver. Although the BAT had excellent specificity (94%) and positive predictive value (94%) when assessing for engaging Hill-Sachs lesions (ie, low false–positive rate for this parameter), poor sensitivity and negative predictive value (43% and 44%, respectively) were determined. The BAT was not predictive for any other bone loss variable analyzed in this study.
